# Woman’s Hospital Medical College

**Published:** 1875-03-15

**Authors:** 


### WOMAN'S HOSPITAL MEDICAL COLLEGE.

THE fifth annual commencement of the above institution was held on the evening of March 2d, at the First M. E. Church. A large audience of ladies and gentlemen were present. The introductory exercises consisted of an organ voluntary, an invocation by the Rev. Dr. Thomas, and a song quartette. Prof. Bartlett gave the usual address on behalf of the faculty. It consisted of a general exposition of the profession, characteristics of habit and talent necessary for success in medicine, and congratulations and advice to the graduating class.

After another song, Prof. Byford, as President of the Board of Trustees, presented, with a brief but appropriate address, diplomas to the graduating class, consisting of the following persons: Miss Sarah A. Brown, Wis.; Miss Lottie Calkins, Ills.; Mrs. Julia N. Marsh, Cal.; Mrs. Sarah H. Stevenson, New York; Mrs. Elizabeth D. Shelton, Kansas; Mrs. M. P. T. Wagstaff, Kansas; Miss Delight J. Wolf, Mich.; Miss Edith A. Root, Ill.

Sarah A. Brown in behalf of the class presented Flint's Physiology to Prof. Mary H. Thompson, which act was nicely acknowledged by the professor.

Mrs. Sarah H. Stevenson read not only an interesting but also an instructive thesis on the "Evolution of the Heart." Commencing with the mere cell, from which she ascended rapidly up through the vegetable kingdom and lower species of animals, to the heart of the mammalia, showing the various steps of development in the *modus operandi* of conveying the nutritive material to all parts of these various living organisms.

The same lady also delivered the class valedictory. It contained more germs of truth and praiseworthy eloquence than we generally hear in such addresses.

A few remarks by Rev. Dr. Thomas closed the exercises.

